# The Role of Thyroid Hormones in Hepatocyte Proliferation and Liver Cancer

**DOI:** 10.3389/fendo.2019.00532

**Published:** 2019-08-30

**Authors:** Fabio Gionfra, Paolo De Vito, Valentina Pallottini, Hung-Yun Lin, Paul J. Davis, Jens Z. Pedersen, Sandra Incerpi

**Affiliations:** ^1^Department of Sciences, University Roma Tre, Rome, Italy; ^2^Department of Biology, University of Rome Tor Vergata, Rome, Italy; ^3^Ph.D. Program for Cancer Molecular Biology and Drug Discovery, College of Medical Science and Technology, Taipei Medical University, Taipei, Taiwan; ^4^Taipei Cancer Center, Taipei Medical University, Taipei, Taiwan; ^5^Pharmaceutical Research Institute, Albany College of Pharmacy and Health Sciences, Rensselaer, NY, United States; ^6^Traditional Herbal Medicine Research Center of Taipei Medical University Hospital, Taipei Medical University, Taipei, Taiwan; ^7^Department of Medicine, Albany Medical College, Albany, NY, United States

**Keywords:** integrin αvβ3, deiodinase, hypothyroidism, tetrac, celiac disease, exosomes, organoids, spheroids

## Abstract

Thyroid hormones T3 and T4 (thyroxine) control a wide variety of effects related to development, differentiation, growth and metabolism, through their interaction with nuclear receptors. But thyroid hormones also produce non-genomic effects that typically start at the plasma membrane and are mediated mainly by integrin αvβ3, although other receptors such as TRα and TRβ are also able to elicit non-genomic responses. In the liver, the effects of thyroid hormones appear to be particularly important. The liver is able to regenerate, but it is subject to pathologies that may lead to cancer, such as fibrosis, cirrhosis, and non-alcoholic fatty liver disease. In addition, cancer cells undergo a reprogramming of their metabolism, resulting in drastic changes such as aerobic glycolysis instead of oxidative phosphorylation. As a consequence, the pyruvate kinase isoform M2, the rate-limiting enzyme of glycolysis, is dysregulated, and this is considered an important factor in tumorigenesis. Redox equilibrium is also important, in fact cancer cells give rise to the production of more reactive oxygen species (ROS) than normal cells. This increase may favor the survival and propagation of cancer cells. We evaluate the possible mechanisms involving the plasma membrane receptor integrin αvβ3 that may lead to cancer progression. Studying diseases that affect the liver and their experimental models may help to unravel the cellular pathways mediated by integrin αvβ3 that can lead to liver cancer. Inhibitors of integrin αvβ3 might represent a future therapeutic tool against liver cancer. We also include information on the possible role of exosomes in liver cancer, as well as on recent strategies such as organoids and spheroids, which may provide a new tool for research, drug discovery, and personalized medicine.

## Introduction

**Figure 1:**
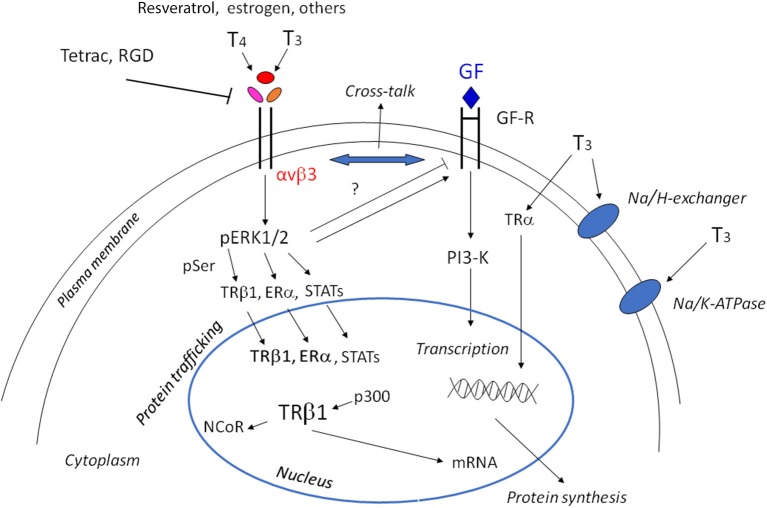
Scheme of non-genomic and genomic actions of thyroid hormones. Non-genomic actions start at the integrin αvβ3, through MAPK/ERK1/2 they may go to the cytoplasm and nucleus to modulate gene expression. The shuttling of the αv monomer to the nucleus is not shown for the sake of simplicity. The cross-talk of thyroid hormones with growth factors is indicated by the double arrow and can be modulated by ERK1/2 activation. The modulation of the membrane Na/K-ATPase pump, either in activation or inhibition depending on the cell type and context, and activation of the Na/H exchanger is also indicated. In the nucleus, interaction of T3 gives rise to the shedding of the corepressor and the interaction with coactivators. Downstream of the activation of TRβ1, Erα, STATS there is the activation of tumor cell proliferation, angiogenesis, and growth factors (GF), but also cytokines through JAK1/2.

Thyroid hormones 3,5,3′-triiodothyronine (T3) and 3,5,3′,5′-tetraiodothyronine (T4) play an essential role in the regulation of cell function during growth, development and metabolism, through two different mechanisms: genomic and non-genomic. The genomic action takes place through the classical nuclear receptors TRα and TRβ, together with modulatory factors such as coactivators and corepressors to regulate gene expression and protein synthesis (1). TRα can stimulate both proliferation and differentiation through β-catenin, while TRβ shows antiproliferative effects in cancer cells and is a differentiation factor. Loss of TRβ is followed by oncogenic transformation. Thyroid hormone receptors and estrogen receptors can cross-talk to modulate physio-pathological responses (2, 3). But thyroid hormones may also give rise to non-genomic effects mediated by integrin αvβ3. These non-genomic effects mainly occur at the plasma membrane level and involve membrane transport systems such as the transporters for glucose and amino acids, the Na^+^/H^+^ exchanger, Na^+^/K^+^-ATPase activity, and kinase activities such as Mitogen-Activated Protein Kinase (MAPK) and Phosphatidyl Inositol 3-Kinase (PI3-K) (4–6), thus increasing angiogenesis and tumor cells proliferation. Integrins are plasma membrane integral proteins that bind extracellular matrix (ECM) proteins such as vitronectin, fibronectin and osteopontin, and regulate cell-cell adhesion (7). Both hormones bind to integrin αvβ3, but T3 binds to the S1 receptor site, activating PI3-K through src, whereas both T3 and T4 bind to a second site S2, leading to the activation of the MAPK pathway and cell proliferation (8). Integrin αvβ3 can also bind other small molecules, such as resveratrol and the steroid hormones (estrogens and androgens) and cancer growth may be modulated by this type of interaction [(9); Figure 1].

## The liver

The liver can be considered privileged from an immunological point of view because it receives 75% of its blood from the portal vein coming from splanchnic districts and about 25% from hepatic artery blood. The major fraction from the portal vein is blood coming from the intestine, stomach, spleen, pancreas, and other organs. Therefore, the portal blood contains components from intestinal uptake of nutrients without the lipid components that go to the lymphatic vessels (10). This special feature of the liver cells makes human hepatocellular carcinoma (HCC) the second leading cause of death in the world.

The liver is composed mainly of hepatocytes, but there is also a small fraction of cells that are important both in physiology and pathology, such as Kupffer cells of the immune system with phagocyte activity, endothelial cells, and Hepatic Stellate Cells. The liver, unlike other tissues in the body, is capable of renewing itself in a very efficient way, and many papers have been published on liver regeneration after partial hepatectomy. The pathways followed in the regenerative process have turned out to be interesting because it is possible in this way to obtain knowledge on cell proliferation, differentiation and tumor growth (11). The first phase of regeneration, called the “priming phase,” prepares the cells to respond to growth factors. The second phase is initiated by the activation of growth factor receptors, and among these the most important appears to be the Epidermal Growth Factor Receptor (EGFR) and c-MET or Hepatocyte Growth Factor Receptor (11). They act in concert until the end of the regenerative process, and then proliferation stops. Important inhibitors of liver regeneration are transforming growth factor-β (TGF-β) and the integrins, which allow the communication between ECM proteins and the cells. Thus, thyroid hormones are important modulators of the regeneration process, because they are able to cross-talk with growth factors such as EGF, TGF-β, and IGF-1 as well as with the integrins, essential players in the mechanism of thyroid hormones (12).

The aim of the present paper is an evaluation of our current knowledge of thyroid hormones in the liver and of the mechanisms related to cell growth and metabolism that may lead to liver cancer. We consider some particular features of liver cells, such as regeneration and the capability to give rise to several metabolic pathologies (fibrosis, cirrhosis, non-alcoholic fatty liver disease). We also consider the possibility that exosomes might modulate thyroid hormone responses in the context of liver cancer, and we provide some information on the frontiers of biotechnology concerning organoids and spheroids.

## Thyroid Hormones and Liver Disease

The liver represents a major target for thyroid hormones, which are involved in the regulation of body weight, lipogenesis, lipid metabolism, and insulin resistance. Therefore, they may have a key role in the pathogenesis of several diseases that affect the liver, such as Alcoholic Liver Disease and non-alcoholic steatohepatitis (NASH), which may evolve into cirrhosis and HCC. Among the thyroid hormone receptors, TRβ is the one mainly expressed in the liver, while TRα is more common in the cardiovascular system and in bone (13). The role of TRβ in mice was demonstrated by the group of Cheng, studying a dominant negative mutation in TRβ (Thrβ^PV/PV^). These mice develop hepatic steatosis within a few months and have significantly larger livers (14). The mutated mice show increased activation of Peroxisome Proliferator–Activated Receptor-γ signaling and decreased fatty acid β-oxidation, leading to lipid accumulation and increased hepatic triglyceride content (14). At variance with this, mice with a mutation in TRα (Thrα^PV/PV^) showed decreased weight and less hepatic lipid accumulation and also decreased lipogenesis.

Thyroid hormones increase the levels of free fatty acids by stimulating lipolysis from dietary fats, although they also stimulate the uptake of free fatty acids by the fatty acid binding protein and fatty acid translocase. The conversion of glucose to fatty acids and *de novo* lipogenesis is stimulated by other hormones and by the diet. Thyroid hormones also regulate the expression and activities of many transcription factors involved in lipogenesis, such as the Sterol Regulatory Element-Binding Protein (SREBP)-1C, liver X receptors and Carbohydrate-Responsive Element-Binding Protein (15). Despite the role of thyroid hormones in *de novo* lipogenesis, they do not increase triglyceride levels but reduce the apolipoprotein B100 and also Very Low-Density Lipoproteins (VLDL) and Low-Density Lipoproteins (LDL) (16). Thyroid hormones also maintain constant sterol levels by modulating all possible pathways of synthesis, export, import, and the conversion to bile acids. In particular, thyroid hormones induce the expression of the limiting enzyme of the cholesterol synthesis, HMG-CoA reductase (17).

Liver fibrosis and cirrhosis are characterized by chronic damage to liver tissue, leading to chronic inflammation, and to altered matrix tissue generation and vascularization. Therefore, the liver progressively looses its functions and this may give rise to the development of cancer. An important role in liver tissue regulation and dysregulation is provided by the ECM proteins that convey information from cell to cell and also from the extracellular to the intracellular compartments. These proteins, which include integrins and collagen, may be important for tissue remodeling and also in the progression of fibrosis, cirrhosis, and cancer (18, 19).

Alcoholic fatty liver disease and non-alcoholic fatty liver disease (NAFLD) represent a major public problem all over the world. Alcohol abuse is the primary cause of several diseases such as fatty liver, alcoholic hepatitis, and cirrhosis (20, 21). Alcohol is metabolized by the liver, which is the primary site of damage. Alcoholic steatohepatitis follows Alcoholic Liver Disease and is characterized by hepatic fat accumulation, infiltration of inflammatory cells, and injury to liver tissue. The process of infiltration by macrophages and neutrophils is mediated by osteopontin produced by the liver. The effects of this protein can be mediated by integrins (22), and osteopontin also appears to be involved in NAFLD/NASH diseases. This cytokine is increased in model systems of these pathologies. Compared to wild type animals, osteopontin-knock-out mice showed decreased liver injury and fibrosis (22). Osteopontin levels are instead increased in some models of liver injury, such as treatment with CCl_4_, although the mechanisms are not clear at present (23).

Epidemiological and clinical reports show an association between NAFLD/NASH and thyroid dysfunction in the form of established or subclinical hypothyroidism. The percentage of hypothyroidism was 15–36% among patients with NAFLD/NASH (20). It has been suggested that NAFLD/NASH are hepatic markers of insulin resistance and metabolic syndrome (24, 25), and insulin resistance can in part be prevented by treating hypothyroidism (20). Among the possible mechanisms proposed is a role of adipocytokines in NAFLD in the presence of hypothyroidism (26). An increased level of leptin has also been reported for hypothyroid patients; this may be responsible for the development of NAFLD/NASH. Leptin is an adipocytokine; it is increased in obesity and may give rise to insulin resistance (27). NAFLD patients show abnormal lipid profiles, with high levels of cholesterol, LDL, and triglycerides. Thyroid hormones acting through the β receptor may cause a reduction of body weight and fat and a decrease in cholesterol and triglyceride levels in hepatocytes (28, 29).

In the context of liver cancer and liver pathologies, the possible effects of oxidative stress, mitochondrial dysfunction, and reactive oxygen species (ROS) production should also be mentioned. Oxidative stress alters the activity of deiodinases, as discussed in the paragraphs that follow, and thyroid hormones can modulate cell function through oxidative stress (30, 31). The role of ROS in thyroid hormone signaling is well-known from the cross-talk between thyroid hormones and the immune system (32, 33).

**Figure 2:**
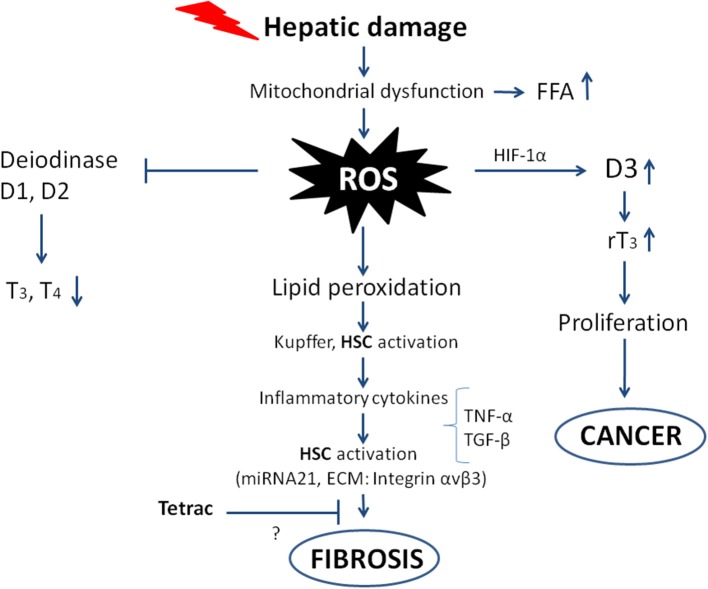
Scheme showing the possible pathways from hepatic damage to fibrosis leading to cancer. The oxidative stress and ROS production follow mitochondrial dysfunction and hepatic damage. The direct consequences are impairment of deiodinase activity leading to decreased T3 production, on the other hand deiodinase 3 is imbalanced with increased activity and increased rT3, which stimulates the proliferation of tumor cells. The ROS produced give rise to inflammatory cytokines that increase the ROS and activate Hepatic Stellate Cells (HSC), leading to fibrosis and eventually cancer. The inhibition by tetrac of the fibrogenic process is only suggested as shown by the question mark, with integrin αvβ3 being among the ECM components involved in the “activation” of the Hepatic Stellate Cells.

Liver fibrosis begins with some damage to liver cells that can be of different nature: physical injury, infection by virus or bacteria (lipopolysaccharides), alcohol, etc. This gives rise to mitochondrial dysfunction and an increase in free fatty acids and ROS, leading to lipid peroxidation, activation of Kupffer cells and Hepatic Stellate Cells. In the case of hepatic injury, expression of the nuclear thyroid hormone receptor in Hepatic Stellate Cells is inhibited, and the dominant hormone receptor becomes TRα, which participates in the fibrogenic response, producing a stronger wound-healing response and higher contractility (34). The levels of inflammatory cytokines increase, causing a further increase in ROS formation, impairment of deiodinase activity, an increase in cell proliferation, and ultimately fibrosis leading to cancer, as will be described in the following sections [(20, 35); Figure 2].

Oxidative markers and inflammation actually appear very early in younger populations as well. A recent paper showed that Ox-LDL and the serum level of Triggering Receptor Expressed on Myeloid cells-1 are associated with cardiovascular risk and other health risks (36). In humans the association of oxidative and inflammatory markers with cholesterol levels, reported for a young healthy population, indicates that it could be very important to start early with an evaluation of these markers, in order to prevent future cardiac pathologies (36). The paper cited does not deal with liver diseases, but it draws attention to the condition of a young human population and their lifestyle. Prevention of some diseases such as liver diseases should start as soon as possible.

In line with the previous topic, we want to recall another pathology affecting more and more children and adolescents: Celiac disease. This is an inflammatory disease of the gut that may develop when persons are exposed to a gluten-containing diet. The intestine as well as the liver are the organs mainly involved, particularly in the young population affected by NAFLD, the most common liver disease in school-age individuals. Very often the diagnosis of Celiac disease precedes that of liver disease (37). Celiac disease is often present in hypothyroid patients, who are more prone to develop cancer and in particular liver cancer (38, 39). Therefore, the development of liver disease and hypothyroidism is becoming more complicated for younger generations.

## Thyroid hormone pathways leading to physio-pathological responses in liver diseases up to cancer

Normal hepatocytes replicate by entering the cell cycle, and in the presence of an injury the process is the same, but it may become dysregulated following conspicuous tissue damage with associated oxidative stress. In any case a regenerative response takes place. In the period of injury-activated regeneration, genes that normally are quiescent become activated through a processes recalling fetal development. Among these processes is the activation of deiodinases, seleno-dependent enzymes that are able to both activate and inactivate thyroid hormone formation in the peripheral tissues (40–42). In particular the levels of deiodinase 3, which hydrolyzes and inactivates both T3 and T4, become upregulated following liver damage and oxidative stress, and the result is a decrease in active T3 levels and increased formation of reverse T3 (rT3) and increased cell proliferation (43–47). Elevated levels of deiodinase 3, mediated by HIF-1, are also reported in both fetal and cancer development (48–50). In liver injury, hepatocytes show a decreased expression of deiodinase 1 and increased levels of deiodinase 3; these variations are regulated by Hedgehog ligands (51). Deiodinase 3 is also more expressed in non-differentiated tissues, such as the developing embryo and cancer (45, 51). Tumor growth or HCC give similar responses to development and injury, and hypothyroidism is associated with a 2- to 3-fold increased risk of cancer development in women. A similar association has not been reported for men (52–54).

Deiodinase 2 is not highly expressed in the liver tissue of an adult, although it is briefly expressed in mouse hepatocytes around birth. This brief appearance seems to be important for the future sensitivity to diet-induced lipid asset for the posttranslational modifications involving DNA methylation and leading to hepatic steatosis, hyperlipidemia and obesity (55). This has been demonstrated by the development of D2-KO mice (ALB-D2KO) with a selective inactivation of deiodinase 2, the resulting phenotype shows resistance to steatosis, hyperlipidemia and obesity. The same researchers also studied the molecular mechanisms involved in this mouse phenotype; the results show that this decreased vulnerability to liver steatosis and diet-induced obesity in the ALB-D2KO mice is due to a reduction in the hepatocyte expression of liver zinc-finger protein-125 (zfp125), a FoxO1-inducible transcriptional repressor responsible for lipid accumulation through a reduced secretion of VLDL. The situation is complicated from both metabolic and hormonal points of view because Forkhead box O1 (FoxO1) is known to be inhibited by insulin, which normally decreases the lipidemia (56).

T3 acts as a mitogen via Protein Kinase A (PKA)/β-catenin activation, leading to activation of cyclin D1 in normal hepatocytes (50). Thyroid hormones have a very complex interaction with their receptors and deiodinases. T3/TR interaction leads to inhibition of the Wnt/β-catenin pathway via Dickkopf Wnt signaling inhibitor 4 (DKK4), resulting in the inhibition of hepatoma cell proliferation (43, 57–59). Hypothyroidism is also associated with human cancer, although contradictory results have been reported relating cancer progression and thyroid hormones (60, 61). For example, primary hypothyroidism has been associated with a decreased risk of breast cancer (61). This effect may depend on non-genomic actions of thyroid hormones because mutant TRs inhibit transactivation activity in glioma and breast cancer (62). In fact, hypothyroidism is involved in different metabolic pathologies, such as obesity, type 2 diabetes, insulin resistance and cancer (60). The impairment of thyroid hormone homeostasis is not considered sufficient for HCC development, but other liver pathologies must be present in order to impair this equilibrium and eventually start a pro-carcinogenic process, such as inflammation, fibrosis or cirrhosis (3, 63, 64).

The downregulation of nuclear thyroid hormone receptors may act as a signal for tumorigenesis, supporting the concept that thyroid hormone receptors inhibit tumorigenesis (3). In fact, a switch from hypo- to hyper-thyroid conditions can be antitumorigenic (60). This clearly indicates that thyroid hormone signaling and thyroid hormone receptors are important for HCC progression (40). In particular T3 seems to have oncosuppressor properties, although it stimulates proliferation in hepatocytes and other cell types, but at the same time it inhibits the growth of hepatoma cells by increasing the time of the G1 phase of the cell cycle. This is related to a decreased expression of the cell cycle mediator cyclin-dependent kinase 2 and cyclin E, and increased gene expression of transforming growth factor TGF-β (65). Other studies confirm these effects of TH receptor β, although contradictory results have also been reported for a human hepatoma cell line (66).

The liver is a major target for thyroid hormones, and in fact, a higher number of mutations of the thyroid hormone receptors α and β have been found in the liver, also in association with the development of liver cancer. However, so far no clear indication has been found, as the situation of the signaling of the thyroid hormone receptor appears to be quite complicated, not only because of the two different typologies of signaling, non-genomic and genomic, but also because non-genomic and genomic effects of thyroid hormones can cross-talk. In addition, TR-α knockout mice are protected from diet-induced hepatic steatosis and hepatic insulin resistance (67). The TRα mutants in HCC act as dominant negative inhibitors in spite of the concentration of T3, impairing gene transcription (3, 68, 69). At variance with these results, the TRβ mutants play a dominant negative effect only at low-intermediate concentrations. In conclusion, TR mutants may have different effects and roles in the development of cancer (60).

TRβ1 can inhibit the nuclear signaling pathways in HCC and breast cancer cells (70). In agreement with these data, a new role for TRβ1 as an anti-metastatic factor has been shown because it inhibits activation of both ERK and PI3K pathways (3, 69, 71). Mutants of TRα1 and TRβ1 from HCC show many alterations from the WT receptors, which indicate that these mutants may act as repressors or activators of specific genes. A similar situation has also recently been shown in the development of renal clear cell carcinoma, and this causes resistance to thyroid hormones (72). In any case, hypothyroidism is associated with the development of cancer in human beings, probably by decreasing apoptosis, while v-ErbA transgenic mice develop liver cancer because v-ErbA may be a dominant-negative receptor (73). As mentioned above, hypothyroidism is also a risk factor for other pathologies, such as NASH (74), and also for viral hepatitis and alcoholic liver disease (52).

The demonstration that v-ErbA can give rise to tumor formation first came from Barlow et al. (73) who created a transgenic mouse with an ectopic expression of v-ErbA. These animals are affected by hypothyroidism, reduced fertility, decreased body weight and abnormal behavior. The male mice also developed hepatocellular carcinoma. v-ErbA has oncogenic potential through its ability to increase the transformation capability of other oncogenes. Parallel studies have also shown that v-ErbA promotes tumorigenesis by interfering with the AP-1 pathway because v-ErbA prevents the inhibition of the AP-1 pathway through thyroid hormone receptors. Estrogens may block the v-ErbA effect and this could explain the protective effects of estrogens toward neoplastic transformation in females. At variance with this, androgens would be permissive toward oncogenic transformation due to v-ErbA (73). Typically, glucocorticoids inhibit the activation of AP-1 and in this way they become potent anti-inflammatory agents (75).

As to the link between viral hepatitis and hypothyroidism, it was found that in patients with chronic hepatitis there was an emergency response shown by the increased levels of thyroperoxidase antibodies (AbTPO), and the subjects positive for AbTPO had a higher risk of hypothyroidism. In cells in culture, HCV infection had a role in thyroid autoimmunity, suggesting an interaction between HCV and the thyroid. At variance with this, patients with chronic hepatitis B virus infection were less prone to autoimmune thyroid disease (76, 77).

Several studies have suggested that thyroid hormones stimulate tumor growth because they stimulate cell proliferation in several types of cancer cells. Thyroid status also affects tumor progression and metastasis both in animals and human beings (60).

Hercbergs et al. showed that hypothyroxinemia can be a compassionate strategy to prolong the life expectancy of terminal tumor patients (78). This is based on a methimazole therapy, to keep low Thyroxine, free T4 and TSH levels, and at the same time to have a normal euthyroid condition through the administration of T3. T3 inhibits tumor cell growth, but an impairment of TH homeostasis alone is not enough to decrease HCC development and invasion. In the liver in particular, HCC develops after a slow progression from liver fibrosis, chronic liver injury and cirrhosis, up to the pre-cancerous alterations with the pre-symptomatic feature being the downregulation of nuclear receptors, TRα and TRβ (3). Therefore, there is no contradiction between the data reported by Hercbergs et al. and the finding of an association between hypothyroidism and liver pathologies such as cancer (3, 78). Actually, among the patients participating in the study of Hercbergs et al. only one was affected by a liver tumor, and not all tissues behave in a similar way. The compassionate therapy reported by Hercbergs et al. that can be induced by either thyroidectomy or pharmacologically (by PTU or methimazole, perhaps also TR-KO) is in agreement with the association between thyroid hormone levels and liver cancer. In fact, hypothyroidism leads to a delay in hepatic regeneration as reported before (79, 80). It is difficult to summarize all the contributions dealing with thyroid hormones and liver cancer development. We refer the reader to an excellent review on the epidemiology of liver cancer (81), and another very good recent review where both *in vitro* studies as well as preclinical and clinical studies report on thyroid hormones and cancer (82).

**Figure 3:**
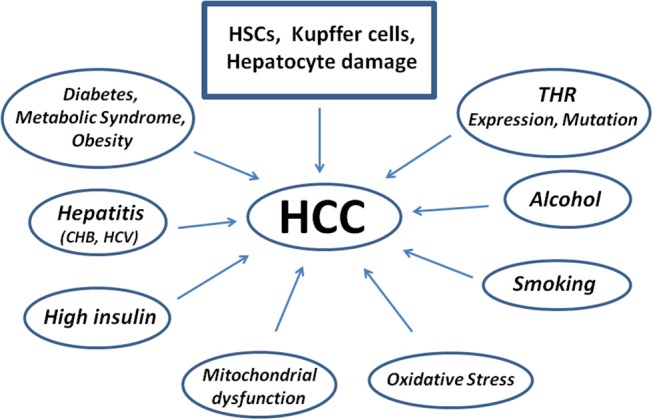
Scheme showing diseases and factors leading to the pathogenesis of Human Hepatocellular Carcinoma starting from Hepatic Stellate Cells damage. Kupffer cells contribute to repair, but may also impair the damage as well as hepatocytes. CHB, Chronic Hepatitis B; HCV, Hepatitis C Virus.

The factors possibly leading to HCC are reported in Figure 3. As to possible genetic markers, microRNA and their dysregulation are genetic factors strongly associated to the pathogenesis of tumor growth and HCC. Exosomes with high levels of miRNA exit the cell and contribute to the spread and invasion of the tumor through activation of the Phosphatase and tensin homolog/3-Phosphoinositide-dependent protein kinase 1/Akt signaling pathway, better known as PTEN/PDK1/Akt (83).

## Metabolism of cancer cells and thyroid hormones: PKM2

The progression of cancer also depends on metabolism. In particular, tumor cells have an increased aerobic glycolysis and lactic acid production; for cancer cells this process is called the Warburg effect (84). It was later found that there is a connection between mitochondria and thyroid hormones in the modulation of this process (85, 86). Suhane and Ramanujan evaluated different metabolic parameters and activities in breast cancer cells, such as lactate generation, oxygen consumption, mitochondrial viability by the MTT assay, and hexokinase activity as the first step of glycolysis. They found that T3 directly increases the metabolism of mitochondria in breast cancer cells and also the expression of one of the isoforms of pyruvate kinase that is responsible for the Warburg effect (85).

Pyruvate kinase catalyzes the last step of glycolysis that converts the phosphoenolpyruvate to pyruvate through the transfer of one phosphate group to ADP (86, 87). Mammals have four isoforms encoded by two genes: PKL is found in liver and other tissues; PKM is present in two isoforms, PKM1 and PKM2, that show the same catalytic activity. PKM2 is more active in regenerating tissue, embryogenesis and cancer, but is also present in non-proliferating and differentiated tissues (88, 89). Therefore, active PKM2 is important for cancer cell metabolism and survival. Cells transformed to express PKM1 instead of PKM2 switched from aerobic glycolysis to mitochondrial respiration and were unable to give rise to tumor formation (90).

Recently Zhao et al. used a xenograft in a murine model to show that PKM2 is able to activate the nuclear transcription factor SREBP-1a, leading to cell proliferation and increased tumor progression. The interaction appears to be highly specific for this type of SREBP-1a (91, 92). This is an important result as it confirms the role of lipid accumulation in cancer progression. Gnoni et al. showed that in HepG2 cells, T3 activates SREBP and the effect is inhibited by tetrac (93).

Hedgehogs (Hh) are a morphogen family that represents an evolutionary highly conserved pathway, from *Drosophila* to human beings. These proteins are able to move from the cell membrane to the nucleus; they have an essential role in embryonic development, and dysregulation of Hh may lead to tumor development (94). The cAMP/PKA pathway is an important negative modulator of the Hh pathway. PKA is important for Sonic Hedgehog (Shh), the main Hh paralog, in fact it phosphorylates Gli (glioma-associated oncogenes) transcription factors repressing gene transcription (95).

Thyroid hormones are tumor suppressors and inhibitors of Shh signaling in Basal Cell Carcinoma. This inhibition may be mediated by deiodinase cross-talk, in particular an increase in deiodinase 3 via Shh/Gli2 leading to a decrease in T3 and increase of rT3, as reported above. Hedgehog-depleted mice show elevated thyroid hormone levels because thyroid hormones are tumor suppressors and inhibitors of Shh signaling in Basal Cell Carcinoma (96). The cAMP/PKA pathway has the opposite effects on Shh signaling. The same Hedgehog pathway is activated in many other pathologies affecting the human liver, including NAFLD and liver fibrosis (51, 97). The increase in deiodinase 3 leads to decreased T3 levels available to modulate gene expression, including the conversion of PKM2 from tetramer conformation to the dimer/monomer conformation that slows cancer progression (88). cAMP (or forskolin) has the opposite effects, activating D2 and therefore the production of T3 from T4 (49, 88). In conclusion, hypothyroidism is a condition that may lead to cancer progression due to rT3 stimulation of cell proliferation, but also because the decrease in T3 leads to an increased activity of the glycolytic pathway typical for cancer cells (88).

## Molecular mechanisms modulated by thyroid hormones, through integrin αvβ3 involved in liver cancer

The family of metalloproteinases consists of more than 20 structurally related, zinc-dependent endopeptidases, that are able to degrade (but also activate) different components of the ECM, such as growth factors, cytokines, and chemokines that reside in the ECM, nowadays considered an important player in cancer progression (98). Through their proteolytic activity they play a role in cancer metastasis and invasion by regulating the signaling pathways involved in cell growth, survival, metastasis and invasion, but also angiogenesis and inflammation (99). Therefore, Matrix Metalloproteinases (MMPs), in particular MMP-2 and MMP-9, are involved in cancer metastasis, and inhibitors of MMP are studied as possible antitumor tools. One of the possible effects is the lysis of the ECM components. Thyroid hormones increase the expression of these MMPs, and a nano-formulation of tetraiodothyroacetic acid, Nano-diamino-tetrac, is able to downregulate the expression of MMP-2 and MMP-9 (100, 101).

The growth of normal tissues, as well as the growth of tumors, depend on the local formation of vasculature, and research on cancer treatment has focused on vascular targets and related growth factors, such as VEGF and basic fibroblasts growth factors (bFGF). Both T3 and T4 are pro-angiogenic as shown when using the chick-egg chorioallantoic membrane model (102). MMP, VEGF, and other angiogenic growth factors acting via miR-126 may be important therapeutic targets in liver cancer (103). In the context of growth factors and cross-talk with thyroid hormones, EGF should also be mentioned because liver cells express high levels of the receptor for this growth factor, which is associated with drug resistance and the angiogenic processes.

Many papers have shown the role of microRNA in cancer progression. MiR-21 and miR-15A play a role in metastasis, but their expression is also modulated by thyroid hormones (101, 104–107) and these effects on cancer progression start at the integrin αvβ3. Tetrac also seems to act effectively on the miRNAs (101); more information on this topic can be found in a very recent review on miRNA in HCC (108).

Thyroid hormones can interact with and modulate the action of growth factors and this may also be related to cancer metastasis. Among growth factors, TGF-β seems to be involved in liver fibrosis and cancer development. TGF-β is a pro-fibrogenic cytokine upregulated in liver disease (109) and apparently there is a direct relationship between thyroid hormones and TGF-β in fibrosis (110). The role of TGF-β in oncogenic transformation has been widely revised and appears to be mediated by the activation of MAPKs and interaction with several types of integrins such as integrin αvβ3 (111). The possible therapeutic approach has also recently been evaluated (112).

Experiments carried out on a pituitary cell line, GH4C1, showed the opposite effect of T3 on the SMAD binding element (SBE) with respect to TGF-β in promoting transcriptional activation of SBE. A more recent paper shows that HEP-G2 treatment with Hexachlorobenzene (HCB), a hormone interferent that gives rise to hypothyroidism, may be reverted by statins through TGF-β, and it is also able to inhibit deiodinase 1, which is highly expressed in the liver, thus decreasing the production of T3 from T4. This could be responsible for the inhibitory effect on tumor promotion caused by statins (and T3 also) (113).

T4 promotes Epithelial Mesenchymal Transition (EMT) through integrin αvβ3, and induction of β-catenin and nanotetrac inhibits this pathway (114). Wnt/β-catenin is a pathway involved in fibrosis and hepatic tumor, as reported above. Wnt signaling inhibits glycogen synthase kinase (GSK-3β), which prevents β-catenin phosphorylation, leading to cytoplasmic accumulation of non-phosphorylated β-catenin that can enter the nucleus to regulate gene expression (114, 115). We have recently shown that tetrac and Nanotetrac downregulate β-catenin and High Mobility Group A2 in colon cancer and the immune checkpoint PD/PD-L1 (106, 116–119). It has been reported by Alvarado et al. (120) that T3, and the agonist GC-1, stimulate cell proliferation in normal hepatocytes, and the effect is dependent on β-catenin activation, Wnt signaling and PKA activation. At the same time, pre-treatment with either T3 or GC-1 after partial hepatectomy leads to a higher increase in cell proliferation with respect to non-treated cells (120). Wnt signaling is also involved in liver fibrogenesis, a recognized risk factor for liver cancer (3, 121). In this case stroma (Hepatic Stellate Cells, macrophages, endothelial cells) activation arising from inflammation due to liver damage in turn leads to increased proliferation and contractility, altered secretion and activity of ECM, leading to a microenvironment that may favor the development of cancer cells (19, 35).

Chemosensitization of cancer cells by tetrac, particularly those resistant to other cancer therapeutic treatments, has been reported. P-glycoprotein (P-gp, MDR1, ABCB1) is a plasma membrane pump that gives rise to the efflux of cancer therapeutic agents. This pump is mainly responsible for the cell chemoresistance in HCC (122, 123). Thyroid hormones are important modulators of this pump by increasing the transcription of *MDR1*, thus increasing the activity of the pump. The mechanism of this stimulation is not yet known in detail, but it is known that thyroid hormones support chemoresistance. Tetrac, instead, increases the retention time of doxorubicin. Thyroid hormones stimulate the Na/H exchanger, the integral plasma membrane protein that exchanges sodium and protons according to the concentration gradient, thus increasing intracellular pH, and tetrac inhibits it, giving rise to cell acidification and inhibition of MDR function and expression (124). Several other factors inhibit the activity of the P-glycoprotein, increasing the retention time of chemotherapeutic agents (i.e., doxorubicin) besides tetrac, osteopontin, VEGF, and calcium channels blockers (124). Most of these effects of thyroid hormones in cancer are blocked by integrin αvβ3 inhibitors (Table 1).

**Table 1:** Mechanisms of reported and possible chemotherapeutic actions of tetrac/Nanotetrac/Nano-diamino-tetrac.

**Action**	**Example**	**Effects**	**References**
Chemosensitization	Efflux of doxorubicin, P-gp effect;	**↓**	(81, 99, 117)
	Efficiency of chemotherapeutic agents	**↑**	(124)
Radiosensitization	Repair of radiation-induced DSB. Radiation-induced activation of integrin αvβ3	**↓**	(101, 125)
Cell survival gene expression	Antiapoptotic genes (*XIAP, MCL-1*)	**↓**	(101, 102)
	Proapoptotic genes (e.g., *CASP2, BC2L14*)	**↑**	(106)
	Stress-defense genes (e.g., *HIF-1α*)	**↓**	(5, 106, 122)
	Oncogene K-ras WT and mutated	**↑**	(126, 127)
Cell cycle	Cyclins and cyclin-dependent protein kinase genes	**↓**	(106)
Growth factors pathways	*EGFR* gene expression and function	**↓**	(102, 122)
	Vascular calcification, ectopic mineralization	**↓**	(102, 106, 107)
	Wnt/β-catenin	**↓**	(106)
Cytokines	IL-1α, IL-1β, IL-6	**↓**	(101, 106)
	IL-11	**↑**	
Chemokines	CXCL2, CXCL3, CX3CL1, CCL20, CCL26, CXCL12	**↓**	(128, 129)
	CXCL10	**↑**	
miRNA	miRNA15A	**↑**	(101, 104, 106)
	miRNA21	**↓**	(105)
Immunotherapy	Immune checkpoint PD-1/PDL-1, HMGA2	**↓**	(116–119)

*Modified from Davis et al. (101). ↑ increase, ↓ decrease*.

## Do Exosomes Have Something to do With Thyroid Hormone's Actions in Liver Cancer?

Exosomes are vesicles, structures derived from cells that are able to modulate intercellular communication. They may contain a wide variety of molecules: cytokines, growth factors and nucleic acids. Exosomes are pivotal elements that make communication between cells easier through “cargos” whose content may change during diseases, particularly cancer, and this can be important to understand the response to disease. The exosomes impact the recipient cell by either epigenetic or translational and transcriptional changes (130). They modulate tumor cell function through apoptosis, differentiation, angiogenesis, or metastasis. The exchange of small molecules such as miRNA is a main object of study, in fact these miRNAs can be biomarkers with a wide range of applications in the management of pathologies such as cancer (131, 132). Modulation of exosomal miRNA represents a target of the personalized medicine intensely pursued nowadays. In Hepatic Stellate Cells treated with exosomes derived from HCC, it was found that the exosomes were able to convert Hepatic Stellate Cells to Cancer Associated Fibroblasts (CAF). Exosomes from HCC, through miRNA-21, were able to activate Hepatic Stellate Cells through the PTEN/PDK1/Akt pathway, thus promoting cancer progression through the secretion of cytokines that stimulated angiogenesis, such as VEGF, MMP-2, and MMP-9 (83).

To our knowledge, modulation of thyroid hormones' effect through exosomes has not been reported on to date. However, elements of the signaling of thyroid hormones, such as the integrin αvβ3 and the already mentioned PKM2 are known. PKM2, as reported above, is important for tumor cell metabolism, helping the switch from oxidative phosphorylation to the glycolytic pathway, typical of tumor cells. The thyroid hormone is an inhibitor of PKM2 that catalyzes the last step of glycolysis producing pyruvate and ATP (88). PKM2 is a tetramer able to activate STAT3 by phosphorylation, and also SNAP-23, important for the secretion of exosomes (133).

Other pathways involving integrin αvβ3 can be modulated by the exosomes, in connection with the delivery of a cargo of Oviductosome (OVS) to modulate sperm capacitation and fertility (134, 135). Integrin αvβ3 and heparan sulfate–proteoglycan in Hepatic Stellate Cells represent new receptors for the exosomes of these cells (134). In prostate cancer the integrin αvβ3 has been proposed as an easy marker of this type of tumor (136).

Embryonic endothelial progenitor cells are able to produce exosomes through stimulation of folliculogenesis in thyroid cells due to the expression of laminin −1α (137). It has been proposed that extracellular vesicles combined with iPSCs (EV-iPSCs) may represent an easy method to slow down or inhibit liver fibrosis. As reported before, there are many mechanisms involved in Hepatic Stellate Cells activation such as cell injury, altered ECM components, immune defense, metabolic dysregulation, infection, and membrane signaling pathways such as kinases and integrin αvβ3. Therefore, possible inhibitors of integrin αvβ3 such as tetrac or Nanotetrac could prevent or inhibit the fibrogenic process (138, 139).

## The Future of Physiology or the Physiology of the Future?

Monolayer cell cultures have long been used to study the physiological and pathological mechanisms of cells and tissues or organs and have also shown their limitations in not being fully comparable to whole tissues, making them a limited model. That is why, over the years, many different strategies have been developed to cultivate cells that more closely resemble the tissue or organ. The technology of 3D cultures has therefore improved, with the idea of being more similar to the structure and physiology of a tissue that is either healthy or cancerous. These models are supposed to overcome the limitation of monolayer cell cultures. Under defined culture conditions, cells may self-assemble into 3D structures called spheroids. But they may also reproduce the embryonic development and give rise to 3D cultures called organoids. Both spheroids and organoids reproduce the morphology and physio-pathological properties of normal and tumor cells, providing new tools for research, drug discovery, and precision medicine. Among the different strategies we mention the one developed and reported by Takebe et al. (140) starting from human induced pluripotent stem cells (iPSCs). For an extensive review on models for development and diseases with organoids see (141). The liver is an extensively studied tissue in the research of biomaterials (141), in particular for the cytotoxicity of drugs and possible drugs (142). The organoids first became popular in the 70s up to the 90s and were first used to study Developmental Biology. Recently, there has been a revival of organoids as 3D structures derived from stem cells made from various organs—a specific cell type and self-organizing in a specific structure (141).

At present the reports showing effects of thyroid hormones on organoids or spheroids are quite limited. An earlier paper on Endocrinology shows the modulation by T3 of the mRNA of 5′-deiodinase reported either in primary hepatocytes or on positively charged dishes for spheroid cultures. The activation by T3 of mRNA D1 was higher in the spheroid structures than in primary hepatocytes and was unaffected by the inhibitor of protein synthesis cycloheximide (143). A few years later the same group showed an increase of responsiveness to T3 in a spheroid culture with respect to isolated hepatocytes, while the different response may involve the Thyroid hormone response element (TRE) complex (144). Another paper in the same years shows that in a 3D collagen gel-culture, thyroid cells easily give rise to folliculogenesis with the proper orientation and polarization, whereas in the usual monolayer that is not possible (145). A paper on recent advances in 3D cell cultures assessing liver physiology and pathology was reported by Calitz et al. (146). For very recent reviews on 2D, 3D, organoids and spheroids see (147, 148) as well as an extensive Chapter on liver culture models by LeCluyse et al. (149). A very interesting work on the construct of hepatocyte aggregation on chitin-based substrates made of butterfly wings opens a new area of study with biomaterials (150).

The effects of thyroid hormones and tetrac mediated by integrin αvβ3 on spheroids made of HuH7 cells integrin αvβ3-negative HCC, compared to mesenchymal stem cells (MSCs) invasion, migration and differentiation has been reported by Schmohl et al. (151) also suggesting a possible therapeutic approach based on tetrac.

## Conclusions

Let us summarize the main information available on the role of thyroid hormones in hepatocyte growth and liver cancer, one of the world's more common fatal diseases, and the research that focuses on therapeutic tools in order to minimize this burden.

A role of thyroid hormones in the pathogenesis of liver cancer has been studied for many years and can be due to thyroid hormones' status, dysregulation of deiodinases, THR mutations or integrin αvβ3 dysregulation.

Tetrac and its derivatives, counteracting many actions of integrin αvβ3, inhibit and prevent many of the weak points of cell metabolism and functions typical of tumor cells, leading to the inhibition of tumor cell growth. This could represent a feasible therapeutic approach for liver cancer as well.

The new strategies of biotechnological research represented by 2D and 3D culture systems, organoids, spheroids and biomaterials, studying the mechanisms relating to thyroid hormones and liver cancer, may represent new frontiers of models in Physiology and Physio-pathology research.

## Author Contributions

FG, PDV, SI, and JZP wrote the manuscript. VP, H-YL, and PJD revised the draft. All the authors approved the manuscript.

### Conflict of Interest Statement

The authors declare that the research was conducted in the absence of any commercial or financial relationships that could be construed as a potential conflict of interest.
